# Triterpenoids and Steroids from *Ganoderma mastoporum* and Their Inhibitory Effects on Superoxide Anion Generation and Elastase Release

**DOI:** 10.3390/molecules181114285

**Published:** 2013-11-19

**Authors:** Tran Dinh Thang, Ping-Chung Kuo, Tsong-Long Hwang, Mei-Lin Yang, Nguyen Thi Bich Ngoc, Tran Thi Ngoc Han, Chi-Wen Lin, Tian-Shung Wu

**Affiliations:** 1 Department of Chemistry, Vinh University, Vinh City, Nghe An 42000, Vietnam; E-Mails: thangtd@vinhuni.edu.vn (T.D.T.); ngockhoahoa@gmail.com (N.T.B.N.); ngochan.tran1990@gmail.com (T.T.N.H.); 2 Department of Biotechnology, National Formosa University, Yunlin 632, Taiwan; E-Mails: pcckuoo@nfu.edu.tw (P.-C.K.); sai_free@yahoo.com.tw (C.-W.L.); 3 Graduate Institute of Natural Products and Chinese Herbal Medicine Research Team, Healthy Aging Research Center, Chang Gung University, Taoyuan 333, Taiwan; E-Mail: htl@mail.cgu.edu.tw; 4 Department of Chemistry, National Cheng Kung University, Tainan 701, Taiwan; E-Mail: L3891104@nckualumni.org.tw

**Keywords:** *Ganoderma*, triterpenoid, steroid, superoxide anion generation, elastase release

## Abstract

The methanol extracts of the fruiting bodies of *Ganoderma mastoporum* collected in Vietnam was purified to afford eight compounds, including three triterpenoids and five steroids. The purified compounds were examined for their inhibitory effects against superoxide anion generation and elastase release. Among the tested compounds, ergosta-4,6,8(14),22-tetraen-3-one (**3**) exhibited the most significant inhibition towards superoxide anion generation and elastase release with IC_50_ values of 2.30 ± 0.38 and 1.94 ± 0.50 µg/mL, respectively.

## 1. Introduction

The genus *Ganoderma* (Polyporaceae) are extensively used as a Chinese remedy for the treatment of tumors [1,2], hepatopathy [3], enhancement of splenic natural killer cell activity, and serum interferon production in mice [4]. Species such as *G. lucidum* attract much attention because it is a well-known source of various therapeutic agents [5,6]. Previous phytochemical investigations of *Ganoderma* genus afforded a series of triterpenoids and steroids [7,8,9,10,11,12,13]. These principles displayed significant cytotoxicity and anti-inflammatory bioactivity [14,15]. *G. mastoporum* is a fungus widely distributed in Southern China, Northern Vietnam, Malaysia, Philippines, and reported to contain sesquiterpenoids [16]. Therefore, additional studies of the phytochemical constituents of *G. mastoporum* and their bioactivities are merited. In our continuous program aimed at the discovery of anti-inflammatory lead drugs from the natural sources, the chemical composition of the fruiting bodies of *G. mastoporum* collected in Vietnam was investigated to search for the bioactive constituents. In the present study, we wished to report the identification of eight compounds, as well as their inhibitory effects on superoxide anion generation and elastase release.

## 2. Results and Discussion

### 2.1. Isolation and Identification of Compounds **1**–**8**

**Figure 1:**
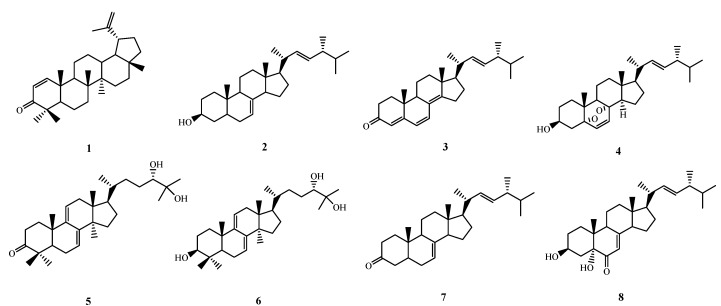
Structures of purified compounds **1**–**8**.

Air-dried and powdered fruiting bodies of *Ganoderma mastoporum* were extracted with methanol, and the combined extracts were concentrated under reduced pressure to give a deep brown syrup. The crude extract was suspended in water and partitioned with ethyl acetate to afford ethyl acetate and water soluble fractions, respectively. Purification of the ethyl acetate fraction by a conventional combination of column chromatographies yielded three triterpenes: Δ^1^-lupenone (**1**) [17], ganodermanondiol (**5**), lucidumol B (**6**) [18], and five steroids: ergosta-7,22-dien-3*β*-ol (**2**) [19], ergosta-4,6,8(14),22-tetraen-3-one (**3**), ergosterol peroxide (**4**) [20], ergosta-7,22-dien-3-one (**7**) [19], and 3*β*,5*α*-dihydroxy-(22*E*,24*R*)-ergosta-7,22-dien-6-one (**8**) [21], respectively. Their chemical structures (Figure 1) were identified by comparison of their physical and spectroscopic data with those reported in the literature.

### 2.2. The Inhibitory Effects of Isolated Compounds on Superoxide Anion Generation and Elastase Release

The purified compounds were subjected to the examination for inhibition of superoxide anion generation and elastase release by human neutrophils in response to *N*-formyl-L-methionyl-leucyl-phenylalanine/cytochalasin B (FMLP/CB) [22], and the data are displayed in Table 1. Only compounds **2** and **8** did not display significant inhibition of superoxide anion generation and elastase release. Other tested compounds **1** and **3**–**7** demonstrated inhibitory effects towards superoxide anion generation and elastase release in a concentration-dependent manner (data not shown). The IC_50_ values were in the range between 2.30 ± 0.38 and 6.36 ± 0.36 μg/mL against superoxide anion generation, and between 1.94 ± 0.50 and 5.01 ± 0.82 µg/mL against elastase release, respectively, compared with the reference compound LY294002 (IC_50_ of 0.40 ± 0.02 and 1.53 ± 0.25 µg/mL towards superoxide anion generation and elastase release). Among the tested compounds, ergosta-4,6,8(14),22-tetraen-3-one (**3**) exhibited the most significant inhibition towards superoxide anion generation and elastase release with IC_50_ of 2.30 ± 0.38 and 1.94 ± 0.50 µg/mL. From the above results, the following structure-bioactivity relationships were deduced: triterpenoids or steroids possessing 3-hydroxy rather than 3-ketone functionalities, such as **2**, **4**, **6**, and **8**, displayed weaker superoxide anion generation inhibitory effects. Comparison of the bioactivity of **2** and **7** also exhibited this tendency. The 24,25-dihydroxy substitutions in **5** and **6**, and the 5-hydroxy group in **8** decreased the inhibition of superoxide anion generation. There is similar tendency in the inhibition of elastase release. However, lucidumol B (**6**) displayed more significant inhibition of elastase release than **5** and **7**.

**Table 1:** Inhibitory effects of purified samples from *G. mastoporum* on superoxide anion generation and elastase release by human neutrophils in response to *N*-formyl-l-methionyl-leucyl-phenylalanine/cytochalasin B (FMLP/CB).

Compound	IC_50_ (µg/mL) ^a^ or (Inh %) ^b^	
	Superoxide Anion Generation	Elastase Release
**1**	3.71 ± 0.79 ***	3.26 ± 0.07 ***
**2**	(34.46 ± 2.42) ***	(27.44 ± 4.90) **
**3**	2.30 ± 0.38 ***	1.94 ± 0.50 ***
**4**	5.28 ± 0.76 ***	(35.99 ± 3.42) ***
**5**	6.36 ± 0.36 ***	5.01 ± 0.82 **
**6**	5.33 ± 0.46 ***	3.32 ± 0.14 ***
**7**	5.02 ± 0.98 ***	4.41 ± 0.50 ***
**8**	(30.11 ± 3.17) ***	(19.46 ± 6.65) *
**LY294002 ^c^**	0.40 ± 0.02 ***	1.53 ± 0.25 ***

^a^ Concentration necessary for 50% inhibition. ^b^ Percentage of inhibition (Inh %) at 10 µg/mL concentration; results are presented as mean ± S.E.M. (n = 3–4); * *p* < 0.05; ** *p* < 0.01; *** *p* < 0.001 compared with the control value. ^c^ A phosphatidylinositol-3-kinase inhibitor was used as a positive control for superoxide anion generation and elastase release.

The formation of superoxide anion in neutrophils can be inhibited by modulating cellular signaling pathways, but also by direct radical scavenging. Therefore the purified constituents **1**–**8** were also subjected to assays of DPPH free radical scavenging activities and ferrous ion chelating capability [23]. None of the examined compounds exhibited significant scavenging of free radicals and capability of chelating ferrous ions at the tested concentration (data not shown). It indicated that the inhibition of superoxide anion generation and elastase release by compounds **1** and **3**–**7** is mediated by modulating cellular signaling pathways [24]. In the previous literature, Δ^1^-lupenone (**1**) and ergosterol peroxide (**4**) were reported to exhibit the inhibitory effects on LPS-induced *i*NOS-dependent NO production in RAW 264.7 cells [25,26,27]. Moreover, ergosta-7,22-dien-3*β*-ol (**2**), ergosterol peroxide (**4**), and ergosta-7,22-dien-3-one (**7**) also inhibited superoxide anion generation release by rat neutrophils [15]. In the present study, the inhibitory effects of these isolated compounds on superoxide anion generation and elastase release by human neutrophils in response to FMLP/CB were reported for the first time.

## 3. Experimental

### 3.1. General Procedure

Melting points were determined using Yanagimoto MP-S3 apparatus (Kyoto, Japan). Optical rotations were measured using a JASCO DIP-370 polarimeter (Oklahoma City, OK, USA). The UV spectra were obtained on a Hitachi UV-3210 spectrophotometer (Tokyo, Japan), and IR spectra were recorded on a Shimadzu FTIR-8501 spectrophotometer (Kyoto, Japan). ^1^H- and ^13^C-NMR, COSY, NOESY, HMQC, and HMBC spectra were obtained on the Bruker Avance III-400 NMR spectrometer (Fremont, CA, USA), with tetramethylsilane (TMS) as internal standard and the chemical shifts were reported in δ values (ppm). The electrospray ionization (ESI) mass spectrum was determined using an Agilent 1200 LC-MSD Trap spectrometer (Santa Clara, CA, USA). Column chromatography (CC) was performed on silica gel (Kieselgel 60, 70–230 mesh and 230–400 mesh, E. Merck, Darmstadt, Germany). Thin layer chromatography (TLC) was conducted on precoated Kieselgel 60 F 254 plates (Merck) and the compounds were visualized by spraying with 10% (v/v) H_2_SO_4_ followed by heating at 110 °C for 10 min.

### 3.2. Fungus Materials

The basidiomycete (*Ganoderma mastoporum* (Mont) Pat.) was collected at the Puhuong National Park of Nghean Province, Vietnam, in November 2010 and identified by Dr. Ngo Anh, Department of Biology, Hue University. A voucher specimen (Vinh-TSWu 20100805) was deposited at the herbarium of the Department of Chemistry, Vinh University.

### 3.3. Extraction and Isolation

The fruiting bodies of *Ganoderma mastoporum* (4.3 Kg) were air-dried, powdered, and extracted with methanol (10 L × 3) at ambient temperature, and the combined extracts were concentrated under reduced pressure to give a deep brown syrup (125 g). The crude extract was suspended in water and partitioned with ethyl acetate to afford ethyl acetate (30 g) and water soluble (95 g) fractions, respectively.

The ethyl acetate soluble extracts were subjected to silica gel column chromatography (CC) with *n*-hexane and acetone gradients (15:1 to 1:1) to afford five fractions. Fractions 1–2 and 4–5 did not display significant triterpenoid and steroid spots monitored by TLC so that they were not further purified. Fraction 3 was a triterpene-rich fraction visualized by spraying with 10% (v/v) H_2_SO_4_ followed by heating at 110 °C for 10 min. It was subjected to silica gel column chromatography eluted with chloroform and methanol gradients (100:1 to 1:1) to afford three subfractions. Subfraction 3-1 was purified by SiO_2_ CC (eluted with chloroform/ethyl acetate 20:1) and further recrystallization of the resulted minor fractions with chloroform/methanol to yield Δ^1^-lupenone (**1**, 4 mg) and ergosta-7,22-dien-3*β*-ol (**2**, 6 mg), respectively. Subfraction 3-2 was repeatedly purified by SiO_2_ CC and preparative TLC (pTLC) (eluted with benzene/ethyl acetate 20:1) to afford ergosta-4,6,8(14),22-tetraen-3-one (**3**, 3 mg), ergosterol peroxide (**4**, 8 mg), and ganodermanondiol (**5**, 2 mg), respectively. Subfraction 3-3 was purified by SiO_2_ CC (eluted with benzene/acetone 50:1) to yield three minor fractions (3-3-1~3-3-3). Fractions 3-3-1 and 3-3-2 were repeatedly purified by SiO_2_ CC and pTLC (eluted with benzene/ethyl acetate 20:1) to afford lucidumol B (**6**, 3 mg), and ergosta-7,22-dien-3-one (**7**, 2 mg), respectively. Fractions 3-3-3 was further recrystallized with chloroform /methanol to result in 3*β*,5*α*-dihydroxy-(22*E*,24*R*)-ergosta-7,22-dien-6-one (**8**, 2 mg).

### 3.4. Determination of Inhibitory Effects on Superoxide Anion Generation and Elastase Release

#### 3.4.1. Preparation of Human Neutrophils

Neutrophils were isolated with a standard method of dextran sedimentation prior to centrifugation in a Ficoll Hypaque gradient and hypotonic lysis of erythrocytes. Blood was drawn from healthy human donors (20–30 years old) by venipuncture into heparin-coated vacutainer tubes, using a protocol approved by the institutional review board at Chang Gung Memorial Hospital [22].

#### 3.4.2. Measurement of Superoxide Anion Generation

The assay of the generation of superoxide anion was based on the SOD-inhibitable reduction of ferricytochrome c [22]. Calculations were based on differences in the reactions with and without SOD (100 U/mL) divided by the extinction coefficient for the reduction of ferricytochrome c (ε = 21.1/mM/10 mm).

#### 3.4.3. Measurement of Elastase Release

Degranulation of azurophilic granules was determined by elastase release as described previously [22]. The results were expressed as the percent of elastase release in the FMLP/CB-activated, drug-free control system.

#### 3.4.4. Assay of Scavenging Activity against Diphenyl Picrylhydrazyl (DPPH) Radical

The DPPH free radical scavenging assay was executed according to the reported method [23]. The DPPH radical scavenging activities of the sample solutions were expressed as inhibition percentage and calculated by using the following equation:

Inhibition percentage (%) = [1 − (absorbance of samples at 517 nm)/(absorbance of control at 517 nm)] × 100 (%).

#### 3.4.5. Chelation of Ferrous Ions

The capability of chelating ferrous ions was determined by the reported method with some modifications [23]. The relative capability of chelating ferrous ions was derived from the formula:

[1 − (sample absorbance at 562 nm)/(control absorbance at 562 nm)] × 100 (%)

#### 3.4.6. Statistical Analysis

Results were expressed as mean ± S.E.M. Computation of 50% inhibitory concentration (IC_50_) was computer-assisted (PHARM/PCS v.4.2). Statistical comparisons were made between groups using Student’s *t* test. Values of *p* less than 0.05 were considered to be statistically significant.

## 4. Conclusion

In summary, three triterpenoids and five steroids from the fruiting bodies of *G. mastoporum* collected in Vietnam were characterized and their inhibition of superoxide anion generation and elastase release by human neutrophils in response to FMLP/cytochalasin B was examined. The results provide evidence for the use of the fruiting bodies of *G. mastoporum* as herbal medicines in the treatment of inflammatory diseases, and these purified principles may be potentially useful in developing new anti-inflammatory therapeutic agents.
